# The epitranscriptome of Vero cells infected with SARS-CoV-2 assessed by direct RNA sequencing reveals m6A pattern changes and DRACH motif biases in viral and cellular RNAs

**DOI:** 10.3389/fcimb.2022.906578

**Published:** 2022-08-16

**Authors:** João H. C. Campos, Gustavo V. Alves, Juliana T. Maricato, Carla T. Braconi, Fernando M. Antoneli, Luiz Mario R. Janini, Marcelo R. S. Briones

**Affiliations:** ^1^ Center for Medical Bioinformatics, Escola Paulista de Medicina, UNIFESP, São Paulo, Brazil; ^2^ Department of Microbiology, Immunology and Parasitology, Escola Paulista de Medicina, UNIFESP, São Paulo, Brazil

**Keywords:** epigenetics, epitranscriptome, RNA methylation, SARS-CoV-2 Genome, m6A, direct RNA sequencing

## Abstract

The epitranscriptomics of the SARS-CoV-2 infected cell reveals its response to viral replication. Among various types of RNA nucleotide modifications, the m6A is the most common and is involved in several crucial processes of RNA intracellular location, maturation, half-life and translatability. This epitranscriptome contains a mixture of viral RNAs and cellular transcripts. In a previous study we presented the analysis of the SARS-CoV-2 RNA m6A methylation based on direct RNA sequencing and characterized DRACH motif mutations in different viral lineages. Here we present the analysis of the m6A transcript methylation of Vero cells (derived from African Green Monkeys) and Calu-3 cells (human) upon infection by SARS-CoV-2 using direct RNA sequencing data. Analysis of these data by nonparametric statistics and two computational methods (m6anet and EpiNano) show that m6A levels are higher in RNAs of infected cells. Functional enrichment analysis reveals increased m6A methylation of transcripts involved in translation, peptide and amine metabolism. This analysis allowed the identification of differentially methylated transcripts and m6A unique sites in the infected cell transcripts. Results here presented indicate that the cell response to viral infection not only changes the levels of mRNAs, as previously shown, but also its epitranscriptional pattern. Also, transcriptome-wide analysis shows strong nucleotide biases in DRACH motifs of cellular transcripts, both in Vero and Calu-3 cells, which use the signature GGACU whereas in viral RNAs the signature is GAACU. We hypothesize that the differences of DRACH motif biases, might force the convergent evolution of the viral genome resulting in better adaptation to target sequence preferences of writer, reader and eraser enzymes. To our knowledge, this is the first report on m6A epitranscriptome of the SARS-CoV-2 infected Vero cells by direct RNA sequencing, which is the *sensu stricto* RNA-seq.

## Introduction

The SARS-CoV-2 has a single-stranded positive-sense RNA genome that replicates exclusively in the cytoplasm of the infected cell. In this cycle, viral RNAs are never synthesized from DNA, and therefore, no viral RNAs are ever produced by transcription (Ricardo-Lax et al., 2021). In the infected cell, the virus generates four RNA species: genomic RNA copies generated by replication (positive sense single-strand), subgenomic RNAs (positive-sense) and the corresponding negative-sense intermediates of both genomic and subgenomic RNAs. (V’kovski et al., 2021). The non-structural proteins are directly translated from the positive single-strand genomic RNA and post-translationally processed. The infected cell contains, therefore, viral RNAs (genomic and subgenomic) and cellular transcripts (tRNAs, rRNAs, mRNAs, lncRNAs, microRNAs, etc…).

Cellular and viral RNAs are subjected to chemical modifications that increase the nucleotide diversity from the four canonical bases to over 150 different bases and nucleotides that add important biological features to RNAs and are necessary for proper biological function. Among these modifications the m6A is the most common (Brocard et al., 2017). Both viral and cellular RNAs are substrates for enzymes that add the methyl group (writers), recognize the signals (readers) and remove the methyl groups (erasers). The m6A methylation is associated with the consensus motif “DRACH”, where the third position is the *N* ^6^-Methyladenosine flanked by D = G or A or U, R = G or A, and H = A or U or C (Bayoumi and Munir, 2021).

Besides the viral RNA synthesis, the infection by viruses alters the gene expression patterns of host cells (Wyler et al., 2021). It is also expected that the viral infection affects the epitranscriptomics landscape of infected cells and the epigenomic pattern of viral RNAs, by modification of RNA nucleotide moieties, such as methylation. Nucleotide residue modifications are essential for proper functionality of viral RNAs and cellular transcripts by regulating RNA stability, cellular localization, translational control, and immune response escape (Gokhale et al., 2016; Li et al., 2021). Until the advent of the Oxford Nanopore technology for direct RNA sequencing (Oxford Nanopore Technologies, Oxford Science Park, Oxford, UK), the sequence of RNAs were obtained by chemical sequencing of very small RNAs (<100 bases) or deduced from cDNA sequencing, improperly called “RNA-seq” (Mardis and McCombie, 2017; Viehweger et al., 2019). Also, base modifications were identified by harsh chemical treatments, low resolution antibody-based techniques or laborious mutated reverse transcriptase synthesis (Kietrys et al., 2017). The Oxford Nanopore direct RNA sequencing, the true RNA-seq, is simple, low-cost technique that readily identifies canonical and modified nucleotide residues, as they are *in vivo*, by use of a properly trained computational neural network. Because the direct RNA sequencing does not employ PCR or any other synthesis, it is free from PCR bias, polymerase errors, phasing and fluorophore crosstalk (Pfeiffer et al., 2018).

The Vero cells are largely used for basic and pre-clinical research. In the course of SARS-CoV-2 Pandemic, these cells were vastly used in pre-clinical tests of a wide range of antiviral drug candidates (Agostini et al., 2019; Schultz et al., 2022), vaccine candidates (Folegatti et al., 2020; López et al., 2021), serving as a main screening method for selection of best candidates to progress on clinical trials. Moreover, the current CoronaVac inactivated virus vaccine (Sinovac Biotech) is produced in this type of cell (Bueno et al., 2021). Also, the gold-standard method to evaluate titles of neutralizing anti-bodies after COVID-19, or vaccination, use Vero cell as reporter (Sadoff et al., 2021; Wheatley et al., 2021), reinforcing the clinical and pharmacological relevance of these cells. It is expected that the Vero cell epitranscriptome might be different from the human Calu-3 cells but not so significantly, regarding the viral response, because of highly conserved genes and regulatory pathways in primates. In other words, humans and other primates share common traits, which supports the wide use of Vero cells in medical and pharmacological techniques of SARS-CoV-2 culturing and testing.

By using the direct RNA sequencing technique, we identified a major type of base methylation, the *N* ^6^-Methyladenosine, or m6A, in viral RNAs from Vero infected cells (Campos et al., 2021). The Vero cell line is derived from the African Green Monkey, or vervet, and is widely used for *in vitro* Coronavirus propagation (Jasinska et al., 2013; Warren et al., 2015). Our results are consistent with previous m6A analysis using “non-direct RNA sequencing” or other studies that used direct RNA-seq although detecting other methylation types, such as 5mC (Kim et al., 2020; Chang et al., 2021; Li et al., 2021; Liu et al., 2021b). Here we extended the viral epigenetic analysis to cellular RNAs of the infected Vero cells and compared to human Calu-3 cells.

## Material and methods

### Samples

Direct RNA sequencing reads from uninfected and infected Vero cells (Clone E6, ATCC^®^ CRL-1586™), and SARS-CoV-2 reads from Vero cell lysates and cell culture supernatants were obtained from (Kim et al., 2020; Taiaroa et al., 2020; Campos et al., 2021). Reads from uninfected, and infected Calu-3 cells, and SARS-CoV-2 reads from Calu-3 cell lysates were obtained from (Chang et al., 2021). The first set of samples used here comes from (Kim et al., 2020), and consists of reads from a sequencing experiment conducted on uninfected Vero cells and another sequencing experiment with Vero cells infected with SARS-CoV-2 (Wuhan-Hu-1 isolate GISAID ID: EPI_ISL_413016). The second set comes from (Taiaroa et al., 2020), and consists of reads from an experiment of infection of Vero cells by SARS-CoV-2 (Wuhan-Hu-1 isolate) and another from viral RNA reads from the supernatant of infected cells. The third sample set sequenced in our laboratory (Campos et al., 2021). Sequencing reads are from Vero cells infected by SARS-CoV-2 (Wuhan-Hu-1 isolate) and reads from viral RNAs of cell culture supernatants. Reads from Calu-3 cells infected by SARS-CoV-2 are from SARS-CoV-2/Australia/VIC01/2020, NCBI: MT007544.1. Sequencing data from uninfected Vero cells (“Vero 24h Control” SRA: SRR13089345) from (Chang et al., 2021) were also used. The reference sequences used were: The African Green Monkey annotated transcripts (Ensembl release 105 - Dec 2021) (Lee et al., 2014; Warren et al., 2015) and the *Homo sapiens* annotated transcripts (Ensembl Release 106 - Apr 2022), and the SARS-CoV-2 reference sequence (NC045512.2) as detailed below.

### Contig assembly by mapping to reference sequences

Raw direct RNA sequencing reads from infected and uninfected Vero cells were obtained from https://doi.org/10.17605/OSF.IO/8F6N9 (Kim et al., 2020), and an additional sample of infected Vero cells and from cell culture supernatant from BioProject Accession PRJNA608224 (Taiaroa et al., 2020). Reads from uninfected and infected Calu-3 cells were obtained by direct RNA sequencing (BioProject Accession PRJNA675370, SRA Accession #SRR13089335 for uninfected Calu-3 cells, and #SRR13089334 for infected Calu-3 cells, and #SRR13089345 for uninfected Vero cells) (Chang et al., 2021). Cell Culture, SARS-CoV-2 Infection, RNA Isolation and Direct RNA Sequencing of the present study were performed as described in (Campos et al., 2021). Direct RNA sequencing and base calling were performed with “High Accuracy Mode” using the Guppy program (v-5.0.11) as described in (Campos et al., 2021) and as described elsewhere for third party data (Kim et al., 2020; Taiaroa et al., 2020; Chang et al., 2021). Reads from SARS-CoV-2 infected Vero cells contained in fastq files were mapped against the *Chlorocebus sabaeus*, or vervet, annotated transcripts fasta file from the genome assembly GCA000409795.2 March 2014, NCBI Genome ID: 13136, NCBI Assembly ID: 132581 and NCBI BioProject ID: 215854 (from Ensembl release 105, accessed in December 2021) (Warren et al., 2015) and the SARS-CoV-2 Wuhan-Hu-1 (NC045512.2) sequence reference using Minimap2 (v-2.21-r1071) (Li, 2018). Bam file reads were sorted and indexed using Samtools (v-1.13) (Li et al., 2009).

### Methylation analysis

The “index” and “eventalign” modules implemented in Nanopolish (v-0.13.2) were used for the resquiggling step. Segmented raw signals generated in the previous step, and contained in the eventalign file, were pre-processed with ‘m6anet-dataprep’ and the predictions of m6A modifications in DRACH motifs were obtained *via* ‘m6anet-run_inference’, algorithms implemented in m6anet program (v-1.0.0) (Hendra et al., 2021). The program EpiNano v.1.2 (Liu et al., 2019; Liu et al., 2021a) was also used for m6A detection, with the Epinano-SVM method (https://github.com/novoalab/EpiNano). The extraction of base-calling error features was performed with the “Epinano_Variants” module, and the m6A predictions were performed on RRACH motifs with the “Epinano_Predict “ script included.

### Analysis of DRACH motifs

To perform a detailed inspection on methylated DRACH motifs - checking for sequence information bias – the consensus sequences were obtained from the variation calling step. This procedure was performed with Longshot (v.0.4.1), with default settings for long reads, and establishing a minimum threshold of read depth for 30x coverage to accept the SNV occurrence (Edge and Bansal, 2019). The resulting VCF files obtained in this step were used to generate consensus sequences by the “bcftools consensus” - BCFTools (v.1.15) (Li, 2011). It was then possible to extract all methylated DRACH motifs, flanked by 5 nucleotides at each end to align and stack them using the Tidyverse package (v.1.3.1) (Wickham et al., 2019). To minimize the occurrence of m6a sites that may represent false positives in Vero cell samples (presence of methylated m6A sites with a reduced probability of predictions as a function of many transcripts/reads) and that could add noise to the analysis, the inspection of the DRACH motifs was performed using sequences containing m6A sites with ≥ 0.8 probability of modification threshold, and coverage ≥ 30x. To reduce the occurrence of false negatives in SARS-CoV-2 sequences with fewer reads, and a smaller genome size, the methylation probability threshold was set at ≥ 0.5. Because some DRACH motifs (including 5 nucleotides at each end) can be located at the coordinate ends of cellular and viral transcripts, and result in truncated sequences, the removal of these sequences was necessary. The analysis of nucleotide biases in DRACH motifs was performed with ggseqlogo package (v.0.1) (Wagih, 2017).

### Functional enrichment analysis

To map transcripts against known functional information sources and detect statistically significant enriched terms, the gProfiler web server was used (https://biit.cs.ut.ee/gprofiler/gost) (Raudvere et al., 2019). Overrepresentation was evaluated in terms of Gene Ontology (GO) or through the Kyoto Encyclopedia of Genes and Genomes (KEGG) pathways (Kanehisa et al., 2019). The main GO categories analyzed were Molecular Function (MF), Biological Process (BP), Cellular Component (CC).

### Statistical test for differential methylation

To compare the distributions of methylated sites per transcript in reads from uninfected and infected cells we performed the Wilcoxon-Mann-Whitney (WMW) test as implemented in R version 4.2.1 as a part of R base (Mann and Whitney, 1947; Fay and Proschan, 2010). This is a nonparametric test to compare differences between two independent groups when they are not normally distributed. Under the null hypothesis H0, the distributions of both groups are identical. The alternative hypothesis H1 is that the distributions are not identical, by detecting a significant difference in the medians. Violin plots were produced with package ggplot2 version 3.3.6. (https://www.rdocumentation.org/packages/ggplot2/versions/3.3.6/topics/ggplot).

## Results

### Distribution of m6A sites in cellular transcripts

Direct RNA sequencing data from four experiments (13 datasets) were used for assembly. One sample was obtained by our group (Campos et al., 2021) and other three samples from (Kim et al., 2020; Taiaroa et al., 2020; Chang et al., 2021). The assembly statistics of these samples are shown in **Table 1**. Samples from Taiaroa et al. were obtained by sequencing on an Oxford Nanopore GridION device (5 flow cells). Reads from Kim et al. were obtained by a 100 ng RNA sample in an Oxford Nanopore MinION device (1 flow cell) with a run for 48 hours whereas the samples from Campos et al. were obtained from a 50 ng RNA sample with a run for 30 hours in an Oxford Nanopore MinION. The differences in number of reads reflect size exclusion in real time basecalling as in Campos et al., 2021, were reads < 500 bases were excluded. Also the RNA preparation used in Campos et al., 2021 favored enrichment of viral RNAs as detailed in the Discussion section below.

**Table 1:** Distribution of read lengths, qualities and mapping in different datasets analyzed in this study.

	Total raw reads	Average percent identity	Fraction of bases aligned	Mean read length	Mean read quality	Median percent identity	Median read length	Median read quality	Number of mapped reads	Read length N50	STDEV read length	Total bases	Total bases aligned
Uninfected Vero (Kim et al., 2020)	1,439,291	89.7	0.8	1,084.3	10.6	90.8	831	10.7	1,045,491	1,371	829.1	1,133,635,322	922,214,398
Infected Vero* * (Kim et al., 2020)	879,679	89.3	0.8	1,059.6	10.4	90.4	798	10.5	189,127	1,333	881.3	200,397,442	160,417,377
Infected Vero (Taiaroa et al., 2020)	680,347	90.1	0.8	1,067.4	10.8	91.3	803	11	390,641	1,408	843	416,982,983	340,053,176
Infected Vero (Campos et al., 2021)	22,601	88.5	0.8	849	11.6	89.1	732	11.7	11,727	874	379.1	9,956,327	8,336,639
SARS-CoV-2 in lysate (Kim et al., 2020)	879,679	90.8	1	2,585.5	10.9	91.6	1,738	11.2	645,942	3,440	2,512.6	1,670,076,092	1,613,107,683
SARS-CoV-2 in lysate (Taiaroa et al., 2020)	680,347	90.8	1	1,827.1	11	91.4	1,577	11.3	210,202	2,575	1,602.7	384,054,811	373,263,396
SARS-CoV-2 in lysate (Campos et al., 2021)	22,601	91.1	1	1,262.8	11.7	91.6	1,128	11.8	7,842	1,602	698.9	9,902,573	9,636,856
SARS-CoV-2/Supernatant (Taiaroa et al., 2020)	430,923	88.8	1	1,083.5	9.9	89.6	811	10.1	18,266	1,515	950.6	19,790,887	19,270,761
SARS-CoV-2/Supernatant (Campos et al., 2021)	1,488,392	89.6	1	1,376.6	10.8	90.2	1,091	11	1,721	1,905	1,103.4	2,369,172	2,271,417
Uninfected Calu-3 (Chang et al., 2021)	952,606	89.5	0.9	1,111.7	10.7	90.4	864	10.8	916,464	1,434	799.9	1,018,788,064	962,906,633
Infected Calu-3 (Chang et al., 2021)	1,068,683	89.6	0.9	1,106.4	10.8	90.4	865	10.9	935,132	1,428	789.9	1,034,668,826	976,315,097
SARS-CoV-2 Calu-3 (Chang et al., 2021)	1,070,290	89.7	0.9	1,653	11	90.5	1,524	11.2	98,204	2,399	1,418	162,327,257	150,141,191
Uninfected Vero Cell (Chang et al., 2021)	1,452,561	88.3	0.8	1,123.1	10.4	89.3	845	10.4	1,262,145	1,473	855.5	1,417,475,246	1,143,645,900

All data are from Vero cells samples except for the three rows indicating samples from Calu-3 cells.

In the uninfected Vero cell, 4228 m6A sites were found and these sites are distributed in 1871 transcripts and 1717 known genes. Most sites were mapped to transcripts encoded in the *VCAN*, *AMOTL2*, and *DNAJB1* genes (**Supplementary Table S1**). The sample infected with SARS-CoV-2 (Kim et al., 2020) has 262 m6A sites mapped to 137 transcripts in 112 known genes, in which *AMOTL2*, *TNFAIP3*, *PLK2* contain the highest number of modifications (**Supplementary Table S2**). The second infected sample (Taiaroa et al., 2020) revealed 1023 sites in 544 transcripts and 488 genes. Genes that contain the largest number of sites are *CA12*, *ARHGAP29*, and *MYC* (**Supplementary Table S3**). The sequencing of infected cells of our group showed a total of 35 m6A sites, in 24 transcripts that map to 14 known genes (**Table 2**) which is consistent with observed in other datasets.

**Table 2:** Distribution of m6A sites in the epitranscriptome of the infected Vero cell sample from (Campos et al., 2021).

Transcript stable ID	Gene coordinate	Gene name	Gene description	Transcript position	Number of reads	Probability of modification
ENSCSAT00000015767.1	28:16195607-16199048	*-*	–	758	31	0.9920354
ENSCSAT00000001773.1	11:86509722-86519010	*LUM*	lumican	1106	49	0.971132
ENSCSAT00000011971.1	22:13362375-13364975	*-*	–	554	40	0.9563033
ENSCSAT00000009109.1	19:7000793-7001757	*MIF*	macrophage migration inhibitory factor	435	33	0.8981868
ENSCSAT00000015767.1	28:16195607-16199048	*-*	–	966	41	0.87318575
ENSCSAT00000008980.1	23:54222838-54238006	*SPARC*	secreted protein acidic and cysteine rich	1997	37	0.8047131
ENSCSAT00000017330.1	20:88010616-88015477	*RPS8*	ribosomal protein S8	1082	37	0.8037766
ENSCSAT00000010859.1	21:54602398-54632780	*COL1A2*	collagen type I alpha 2 chain	3830	43	0.77824545
ENSCSAT00000011971.1	22:13362375-13364975	*-*	–	546	31	0.770081
ENSCSAT00000007846.1	15:68596968-68599389	*-*	–	685	33	0.7656064
ENSCSAT00000005189.1	26:48105216-48110896	*ACTC1*	actin alpha cardiac muscle 1	1632	30	0.73731476
ENSCSAT00000000028.1	MT:10751-12128	*MT-ND4*	mitochondrially encoded NADH:ubiquinone oxidoreductase core subunit 4	960	31	0.733373
ENSCSAT00000007475.1	12:60353817-60357759	*-*	–	420	46	0.7216429
ENSCSAT00000009292.1	5:1859454-1862305	*RPS2*	ribosomal protein S2	667	99	0.71115077
ENSCSAT00000011687.1	8:68898693-68901812	*RPL7*	ribosomal protein L7	998	34	0.7100177
ENSCSAT00000010859.1	21:54602398-54632780	*COL1A2*	collagen type I alpha 2 chain	4270	36	0.6952559
ENSCSAT00000013675.1	21:14525637-14528513	*MYL7*	myosin light chain 7	730	43	0.6849283
ENSCSAT00000018059.1	12:21401910-21402595	*-*	–	675	42	0.6834394
ENSCSAT00000011706.1	26:14002075-14004474	*RPLP1*	ribosomal protein lateral stalk subunit P1	198	53	0.66291857
ENSCSAT00000001773.1	11:86509722-86519010	*LUM*	lumican	654	45	0.6566237
ENSCSAT00000013859.1	21:12905280-12914205	*IGFBP3*	insulin like growth factor binding protein 3	2371	31	0.63804483
ENSCSAT00000006068.1	23:82798085-82805936	*RACK1*	receptor for activated C kinase 1	1250	40	0.6353171
ENSCSAT00000018668.1	18:70826193-70826794	*-*	–	218	41	0.60119855
ENSCSAT00000000697.1	16:66963460-66968731	*-*	–	783	34	0.59995514
ENSCSAT00000010859.1	21:54602398-54632780	*COL1A2*	collagen type I alpha 2 chain	3800	39	0.58406496
ENSCSAT00000011706.1	26:14002075-14004474	*RPLP1*	ribosomal protein lateral stalk subunit P1	513	56	0.58338195
ENSCSAT00000013859.1	21:12905280-12914205	*IGFBP3*	insulin like growth factor binding protein 3	2053	38	0.5830461
ENSCSAT00000018059.1	12:21401910-21402595	*-*	–	75	49	0.57314473
ENSCSAT00000008980.1	23:54222838-54238006	*SPARC*	secreted protein acidic and cysteine rich	1943	38	0.5681411
ENSCSAT00000016933.1	6:50663431-50670230	*RPS5*	ribosomal protein S5	398	45	0.55939466
ENSCSAT00000019107.1	16:59453767-59454246	*-*	–	8	49	0.5383941
ENSCSAT00000006068.1	23:82798085-82805936	*RACK1*	receptor for activated C kinase 1	1030	37	0.537564
ENSCSAT00000013016.1	4:76392625-76396331	*-*	–	275	43	0.52486014
ENSCSAT00000000555.1	6:42666353-42671124	*-*	–	1015	90	0.5181154
ENSCSAT00000013675.1	21:14525637-14528513	*MYL7*	myosin light chain 7	519	43	0.50369656

“-” indicates non-annotated genes in the reference genome.

The comparison of infected Vero cells from the studies by (Kim et al., 2020; Taiaroa et al., 2020), shows that they share 143 m6A sites. The *MYC*, *PLK2*, and *PRSS23* genes, for example, share the same 7 methylation sites in each gene (**Supplementary Table S4**). When we observed samples of infected Vero cells from (Kim et al., 2020), and the sample sequenced by our group, we found 6 m6A sites common to both, distributed over 4 transcripts, one of which mapped to the *RACK1* gene (**Supplementary Table S5**). The samples of infected Vero cells from the study (Taiaroa et al., 2020), and from our sample, share 5 m6A sites, distributed over 4 transcripts (**Supplementary Table S6**). All infected samples share only 3 m6A sites, located in 2 transcripts from unidentified genes, namely transcript IDs ENSCSAT00000015767.1 (m6A positions 758, coverage 604, and 966, coverage 787) and ENSCSAT00000011971.1 (m6A position 554, coverage 78) (**Supplementary Table S7**).

The alignment of reads, obtained from the infected Vero cell lysate from the (Kim et al., 2020), to the SARS-CoV-2 reference sequence, revealed 13 m6A sites, 12 of which map to ORF1ab (**Supplementary Table S8**). The SARS-CoV-2 epigenome from the sample sequenced by (Taiaroa et al., 2020), revealed 7 m6A sites, 4 of which are also located in ORF1ab (**Supplementary Table S9**). The SARS-CoV-2 epigenome from the cell lysate in our data showed the presence of 5 m6A sites, two of which located in ORF7b, and 2 located in the N gene (**Supplementary Table S10**). SARS-CoV-2 epigenomes from samples from (Kim et al., 2020; Taiaroa et al., 2020), share 4 m6A sites, 3 of which are in ORF1ab (**Supplementary Table S11**). The epigenomes of the three samples obtained from Vero cell lysates share only one m6A site (**Supplementary Table S12**).

Because the sample set of (Kim et al., 2020) contained the largest number of mapped reads (**Table 1**) this dataset was used to identify differentially methylated transcripts in the Vero cell infected with SARS-CoV-2 (**Table 3**). This analysis shows 55 differentially methylated sites distributed in 21 known genes. Among identified transcripts, are the Tensin 3 (*TNS3*), *NUAK2*, and *METTL9* transcripts whose biological functions are relevant. Also, this same dataset allowed the identification of m6A sites unique to transcripts of the infected Vero cells (**Table 4**). This analysis reveals 47 sites distributed in 37 known genes with attention to the kinase *NUAK2*, Tensin 3 (*TNS3*), Ras member B (*RHOB*) and *METTL9*.

**Table 3:** Differentially methylated known genes obtained by comparison of uninfected cell (U) and infected cell (I) datasets from (Kim et al., 2020).

Transcript stable ID	Gene coordinate	Gene name	Gene description	Position (U)	Reads (U)	Prob. (U)	Position (I)	Reads (I)	Prob. (I)
ENSCSAT00000000076.1	11:118913078-118928281	*TMED2*	transmembrane p24 trafficking protein 2	884	189	0.90227556	1394	33	0.80859786
ENSCSAT00000000076.1	11:118913078-118928281	*TMED2*	transmembrane p24 trafficking protein 2	1703	251	0.8100335	–	–	–
ENSCSAT00000000890.1	6:41640636-41650080	*KDELR1*	KDEL endoplasmic reticulum protein retention receptor 1	1107	237	0.8039204	1640	38	0.8297022
ENSCSAT00000001690.1	1:77213836-77324875	*PICALM*	phosphatidylinositol binding clathrin assembly protein	173	49	0.8412698	895	33	0.8023325
ENSCSAT00000001942.1	1:66788240-66799072	*SERPINH1*	serpin family H member 1	1561	224	0.96509045	2082	31	0.94127834
ENSCSAT00000001942.1	1:66788240-66799072	*SERPINH1*	serpin family H member 1	436	87	0.8628933	–	–	–
ENSCSAT00000001942.1	1:66788240-66799072	*SERPINH1*	serpin family H member 1	1233	178	0.8468479	–	–	–
ENSCSAT00000002495.1	7:30805749-30810720	*HNRNPDL*	heterogeneous nuclear ribonucleoprotein D like	2078	30	0.9407665	1576	68	0.85807246
ENSCSAT00000003445.1	9:95672648-95689247	*TRIM8*	tripartite motif containing 8	1412	46	0.9639772	2315	31	0.83026695
ENSCSAT00000003445.1	9:95672648-95689247	*TRIM8*	tripartite motif containing 8	1568	88	0.94590265	–	–	–
ENSCSAT00000003445.1	9:95672648-95689247	*TRIM8*	tripartite motif containing 8	1486	69	0.90411556	–	–	–
ENSCSAT00000003903.1	16:33752556-33799102	*CLTC*	clathrin heavy chain	3347	241	0.8372275	4409	40	0.8006732
ENSCSAT00000003903.1	16:33752556-33799102	*CLTC*	clathrin heavy chain	252	139	0.8231322	–	–	–
ENSCSAT00000004018.1	16:31833160-31900963	*PPM1D*	protein phosphatase, Mg2+/Mn2+ dependent 1D	870	76	0.9798185	2058	42	0.87876064
ENSCSAT00000004018.1	16:31833160-31900963	*PPM1D*	protein phosphatase, Mg2+/Mn2+ dependent 1D	–	–	–	1657	37	0.8740219
ENSCSAT00000004018.1	16:31833160-31900963	*PPM1D*	protein phosphatase, Mg2+/Mn2+ dependent 1D	1396	108	0.8597186	–	–	–
ENSCSAT00000004018.1	16:31833160-31900963	*PPM1D*	protein phosphatase, Mg2+/Mn2+ dependent 1D	2907	34	0.83353394	–	–	–
ENSCSAT00000004698.1	10:117477904-117486774	*NCL*	nucleolin	2309	274	0.8647666	2014	33	0.87197196
ENSCSAT00000005041.1	5:20128176-20175564	*METTL9*	methyltransferase like 9	847	248	0.801356	992	32	0.8637458
ENSCSAT00000005327.1	14:96801349-96828716	*PDIA6*	protein disulfide isomerase family A member 6	471	290	0.90395755	1509	50	0.8532138
ENSCSAT00000005327.1	14:96801349-96828716	*PDIA6*	protein disulfide isomerase family A member 6	1723	490	0.8176958	–	–	–
ENSCSAT00000006347.1	17:41712528-41714126	*IER3*	immediate early response 3	623	37	0.994408	479	30	0.96617603
ENSCSAT00000006347.1	17:41712528-41714126	*IER3*	immediate early response 3	–	–	–	916	34	0.8894909
ENSCSAT00000006347.1	17:41712528-41714126	*IER3*	immediate early response 3	–	–	–	463	30	0.8717268
ENSCSAT00000006347.1	17:41712528-41714126	*IER3*	immediate early response 3	–	–	–	940	38	0.809437
ENSCSAT00000006347.1	17:41712528-41714126	*IER3*	immediate early response 3	686	65	0.970193	–	–	–
ENSCSAT00000008192.1	8:122282630-122288384	*MYC*	MYC proto-oncogene, bHLH transcription factor	1336	38	0.9933568	2231	42	0.8794924
ENSCSAT00000008192.1	8:122282630-122288384	*MYC*	MYC proto-oncogene, bHLH transcription factor	2248	89	0.8645151	–	–	–
ENSCSAT00000009503.1	9:32202204-32258154	*ITGB1*	integrin subunit beta 1	758	338	0.80759764	1725	55	0.8165182
ENSCSAT00000012714.1	25:24016930-24036641	*NUAK2*	NUAK family kinase 2	2434	39	0.99647045	3301	30	0.95335376
ENSCSAT00000012714.1	25:24016930-24036641	*NUAK2*	NUAK family kinase 2	–	–	–	3136	34	0.82845277
ENSCSAT00000012714.1	25:24016930-24036641	*NUAK2*	NUAK family kinase 2	3165	90	0.9791137	–	–	–
ENSCSAT00000012714.1	25:24016930-24036641	*NUAK2*	NUAK family kinase 2	2372	37	0.93598187	–	–	–
ENSCSAT00000012714.1	25:24016930-24036641	*NUAK2*	NUAK family kinase 2	3394	71	0.86949	–	–	–
ENSCSAT00000013859.1	21:12905280-12914205	*IGFBP3*	insulin like growth factor binding protein 3	1093	343	0.8282119	1378	404	0.8150883
ENSCSAT00000013875.1	21:11292788-11554933	*TNS3*	tensin 3	5866	69	0.9969953	7534	31	0.884178
ENSCSAT00000013875.1	21:11292788-11554933	*TNS3*	tensin 3	5434	41	0.984392	–	–	–
ENSCSAT00000013875.1	21:11292788-11554933	*TNS3*	tensin 3	5226	39	0.98384225	–	–	–
ENSCSAT00000013875.1	21:11292788-11554933	*TNS3*	tensin 3	5129	38	0.98288566	–	–	–
ENSCSAT00000013875.1	21:11292788-11554933	*TNS3*	tensin 3	6065	61	0.9710264	–	–	–
ENSCSAT00000013875.1	21:11292788-11554933	*TNS3*	tensin 3	6453	81	0.9707899	–	–	–
ENSCSAT00000013875.1	21:11292788-11554933	*TNS3*	tensin 3	6145	73	0.96241	–	–	–
ENSCSAT00000013875.1	21:11292788-11554933	*TNS3*	tensin 3	5332	43	0.91376925	–	–	–
ENSCSAT00000013875.1	21:11292788-11554933	*TNS3*	tensin 3	5659	62	0.8688911	–	–	–
ENSCSAT00000013875.1	21:11292788-11554933	*TNS3*	tensin 3	6577	99	0.80982953	–	–	–
ENSCSAT00000017358.1	20:90002030-90008033	*SLC2A1*	solute carrier family 2 member 1	2060	114	0.98355216	2315	32	0.93140286
ENSCSAT00000017358.1	20:90002030-90008033	*SLC2A1*	solute carrier family 2 member 1	2302	173	0.98312795	–	–	–
ENSCSAT00000017358.1	20:90002030-90008033	*SLC2A1*	solute carrier family 2 member 1	1977	135	0.94395334	–	–	–
ENSCSAT00000017358.1	20:90002030-90008033	*SLC2A1*	solute carrier family 2 member 1	2010	156	0.89053315	–	–	–
ENSCSAT00000017358.1	20:90002030-90008033	*SLC2A1*	solute carrier family 2 member 1	2093	173	0.8239867	–	–	–
ENSCSAT00000017703.1	20:116843218-116863010	*EFHD2*	EF-hand domain family member D2	232	39	0.8788946	1959	40	0.8244804
ENSCSAT00000018156.1	14:87249005-87249592	*RHOB*	ras homolog family member B	260	71	0.8870451	122	31	0.8521231
ENSCSAT00000018156.1	14:87249005-87249592	*RHOB*	ras homolog family member B	359	167	0.8001116	–	–	–
ENSCSAT00000018928.1	6:11479407-11480450	*JUNB*	JunB proto-oncogene, AP-1 transcription factor subunit	1032	89	0.9923255	99	36	0.9681243
ENSCSAT00000018928.1	6:11479407-11480450	*JUNB*	JunB proto-oncogene, AP-1 transcription factor subunit	–	–	–	893	60	0.9514262

Reads = Coverage and Prob. – Probability of m6A methylation as calculated by m6anet program.

**Table 4:** Unique m6A sites in known genes of the infected sample – obtained by comparison of uninfected and infected cells dataset (Kim et al., 2020).

Transcript stable ID	Gene coordinate	Gene name	Gene description	Transcript position	Number of reads	Probability of modification
ENSCSAT00000000076.1	11:118913078-118928281	*TMED2*	transmembrane p24 trafficking protein 2	1394	33	0.80859786
ENSCSAT00000000813.1	6:42108267-42112471	*PPP1R15A*	protein phosphatase 1 regulatory subunit 15A	1760	30	0.85488987
ENSCSAT00000000813.1	6:42108267-42112471	*PPP1R15A*	protein phosphatase 1 regulatory subunit 15A	2022	35	0.8545834
ENSCSAT00000000890.1	6:41640636-41650080	*KDELR1*	KDEL endoplasmic reticulum protein retention receptor 1	1640	38	0.8297022
ENSCSAT00000001690.1	1:77213836-77324875	*PICALM*	phosphatidylinositol binding clathrin assembly protein	895	33	0.8023325
ENSCSAT00000001942.1	1:66788240-66799072	*SERPINH1*	serpin family H member 1	2082	31	0.94127834
ENSCSAT00000002458.1	1:56799838-56813650	*EIF3F*	eukaryotic translation initiation factor 3 subunit F	503	44	0.80193645
ENSCSAT00000002495.1	7:30805749-30810720	*HNRNPDL*	heterogeneous nuclear ribonucleoprotein D like	1576	68	0.85807246
ENSCSAT00000003445.1	9:95672648-95689247	*TRIM8*	tripartite motif containing 8	2315	31	0.83026695
ENSCSAT00000003903.1	16:33752556-33799102	*CLTC*	clathrin heavy chain	4409	40	0.8006732
ENSCSAT00000004018.1	16:31833160-31900963	*PPM1D*	protein phosphatase, Mg2+/Mn2+ dependent 1D	2058	42	0.87876064
ENSCSAT00000004018.1	16:31833160-31900963	*PPM1D*	protein phosphatase, Mg2+/Mn2+ dependent 1D	1657	37	0.8740219
ENSCSAT00000004351.1	5:26221337-26225083	*TUFM*	Tu translation elongation factor, mitochondrial	1336	35	0.8196384
ENSCSAT00000004698.1	10:117477904-117486774	*NCL*	nucleolin	2014	33	0.87197196
ENSCSAT00000005041.1	5:20128176-20175564	*METTL9*	methyltransferase like 9	992	32	0.8637458
ENSCSAT00000005327.1	14:96801349-96828716	*PDIA6*	protein disulfide isomerase family A member 6	1509	50	0.8532138
ENSCSAT00000006347.1	17:41712528-41714126	*IER3*	immediate early response 3	479	30	0.96617603
ENSCSAT00000006347.1	17:41712528-41714126	*IER3*	immediate early response 3	916	34	0.8894909
ENSCSAT00000006347.1	17:41712528-41714126	*IER3*	immediate early response 3	463	30	0.8717268
ENSCSAT00000006347.1	17:41712528-41714126	*IER3*	immediate early response 3	940	38	0.809437
ENSCSAT00000006678.1	22:52391813-52417575	*RPN1*	ribophorin I	1615	36	0.8343291
ENSCSAT00000007637.1	23:75149710-75200192	*ATP6V0E1*	ATPase H+ transporting V0 subunit e1	845	44	0.8655202
ENSCSAT00000007678.1	23:74929863-74934532	*DUSP1*	dual specificity phosphatase 1	1649	49	0.9319972
ENSCSAT00000007678.1	23:74929863-74934532	*DUSP1*	dual specificity phosphatase 1	870	33	0.86039066
ENSCSAT00000007678.1	23:74929863-74934532	*DUSP1*	dual specificity phosphatase 1	1281	41	0.80852
ENSCSAT00000008192.1	8:122282630-122288384	*MYC*	MYC proto-oncogene, bHLH transcription factor	2231	42	0.8794924
ENSCSAT00000009011.1	24:72156784-72163500	*SERPINA1*	serpin family A member 1	1109	40	0.84530336
ENSCSAT00000009503.1	9:32202204-32258154	*ITGB1*	integrin subunit beta 1	1725	55	0.8165182
ENSCSAT00000011100.1	6:309506-321785	*BSG*	basigin (Ok blood group)	1931	31	0.90602887
ENSCSAT00000011100.1	6:309506-321785	*BSG*	basigin (Ok blood group)	1863	30	0.81299096
ENSCSAT00000012419.1	4:90497754-90605481	*CAST*	calpastatin	1944	60	0.8325281
ENSCSAT00000012714.1	25:24016930-24036641	*NUAK2*	NUAK family kinase 2	3301	30	0.95335376
ENSCSAT00000012714.1	25:24016930-24036641	*NUAK2*	NUAK family kinase 2	3136	34	0.82845277
ENSCSAT00000012734.1	14:45086920-45111271	*CCT4*	chaperonin containing TCP1 subunit 4	685	55	0.8591496
ENSCSAT00000013859.1	21:12905280-12914205	*IGFBP3*	insulin like growth factor binding protein 3	1378	404	0.8150883
ENSCSAT00000013875.1	21:11292788-11554933	*TNS3*	tensin 3	7534	31	0.884178
ENSCSAT00000014245.1	4:54629194-54636032	*PLK2*	polo like kinase 2	210	36	0.8035949
ENSCSAT00000015796.1	28:15672442-15694148	*KDELR2*	KDEL endoplasmic reticulum protein retention receptor 2	790	50	0.83011115
ENSCSAT00000015906.1	28:13491483-13519507	*CYP3A5*	cytochrome P450 family 3 subfamily A member 5	2297	52	0.81895936
ENSCSAT00000016112.1	28:12047529-12060202	*SERPINE1*	serpin family E member 1	2083	139	0.8208885
ENSCSAT00000017104.1	20:47783935-47785997	*CCN1*	cellular communication network factor 1	411	104	0.8570591
ENSCSAT00000017358.1	20:90002030-90008033	*SLC2A1*	solute carrier family 2 member 1	2315	32	0.93140286
ENSCSAT00000017703.1	20:116843218-116863010	*EFHD2*	EF-hand domain family member D2	1959	40	0.8244804
ENSCSAT00000018156.1	14:87249005-87249592	*RHOB*	ras homolog family member B	122	31	0.8521231
ENSCSAT00000018928.1	6:11479407-11480450	*JUNB*	JunB proto-oncogene, AP-1 transcription factor subunit	99	36	0.9681243
ENSCSAT00000018928.1	6:11479407-11480450	*JUNB*	JunB proto-oncogene, AP-1 transcription factor subunit	893	60	0.9514262
ENSCSAT00000019531.1	14:37013880-37014947	*PCBP1*	poly(rC) binding protein 1	866	58	0.90854704

**Figure 1:**
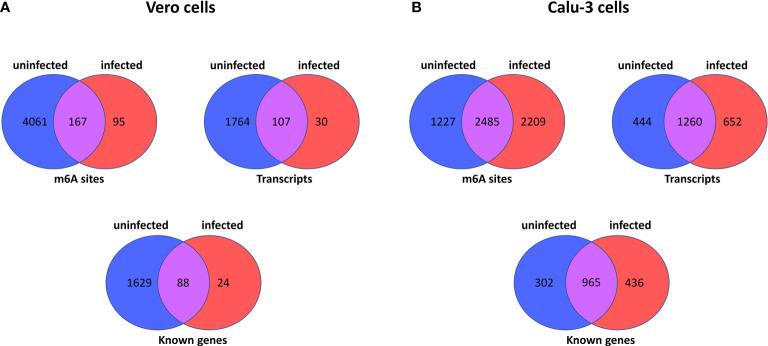
Summary of number of m6A sites, number of transcripts and number of known genes in host cell epitranscriptome data here analysed. The Venn diagrams show the number of m6A sites in uninfected and infected Vero cells (derived from African Green Monkeys) (Kim et al., 2020) **(A)**, and between uninfected and infected Calu-3 cells (human derived) (Chang et al., 2021) **(B)**. In each panel, the number of m6A sites, transcripts and known genes are displayed for each host cell type.

The comparison of Vero cells data with Calu-3 reveals very similar pattern of transcripts and m6A methylation sites. **Figure 1** outlines a set of comparisons made between Vero or Calu-3 cells, by m6A site, transcripts and known genes. Regarding the latter cell type, the **Supplementary Tables S13, S14** show which sites are present in one sample or another (uninfected and infected cells), which sites are common to both (**Supplementary Table S15**), data from (Chang et al., 2021), or which sites are unique to each sample (**Supplementary Tables S16, S17**) (sample from Chang et al., 2021). Genes *SDC1*, *PMM2*, and *SERPINA1* have the greatest number of unique m6A sites in the uninfected cell (**Supplementary Table S17**). In the infected cell, the unique sites are found mainly in the *IFIT2*, *OASL* and *IFIT3* genes (40, 29 and 27 sites respectively) (**Supplementary Tables S16**, **S17**).

Quantitative analysis of differences in m6A methylation between uninfected and infected Vero cells was performed using the Wilcoxon-Mann-Whiteney test with two independent uninfected datasets and two independent infected datasets: (1) Kim et al., 2020 (Uninfected Vero) and Chang et al., 2021 (Uninfected Vero 24h); (2) Kim et al., 2020 (Infected Vero) and Taiaroa et al., 2020 (Infected Vero). For each dataset, the differentially methylated transcripts were filtered by two criteria: (a) there were more than 30 reads supporting the methylated site, and (b) the probability of modification being true was higher than 0.99. The number of methylated sites per transcript type was computed considering the quantity *S*=log(methylated sie/transcript), the (base 10) logarithm of the proportion of methylated sites per transcript, for all transcript types in each dataset.

**Figure 2:**
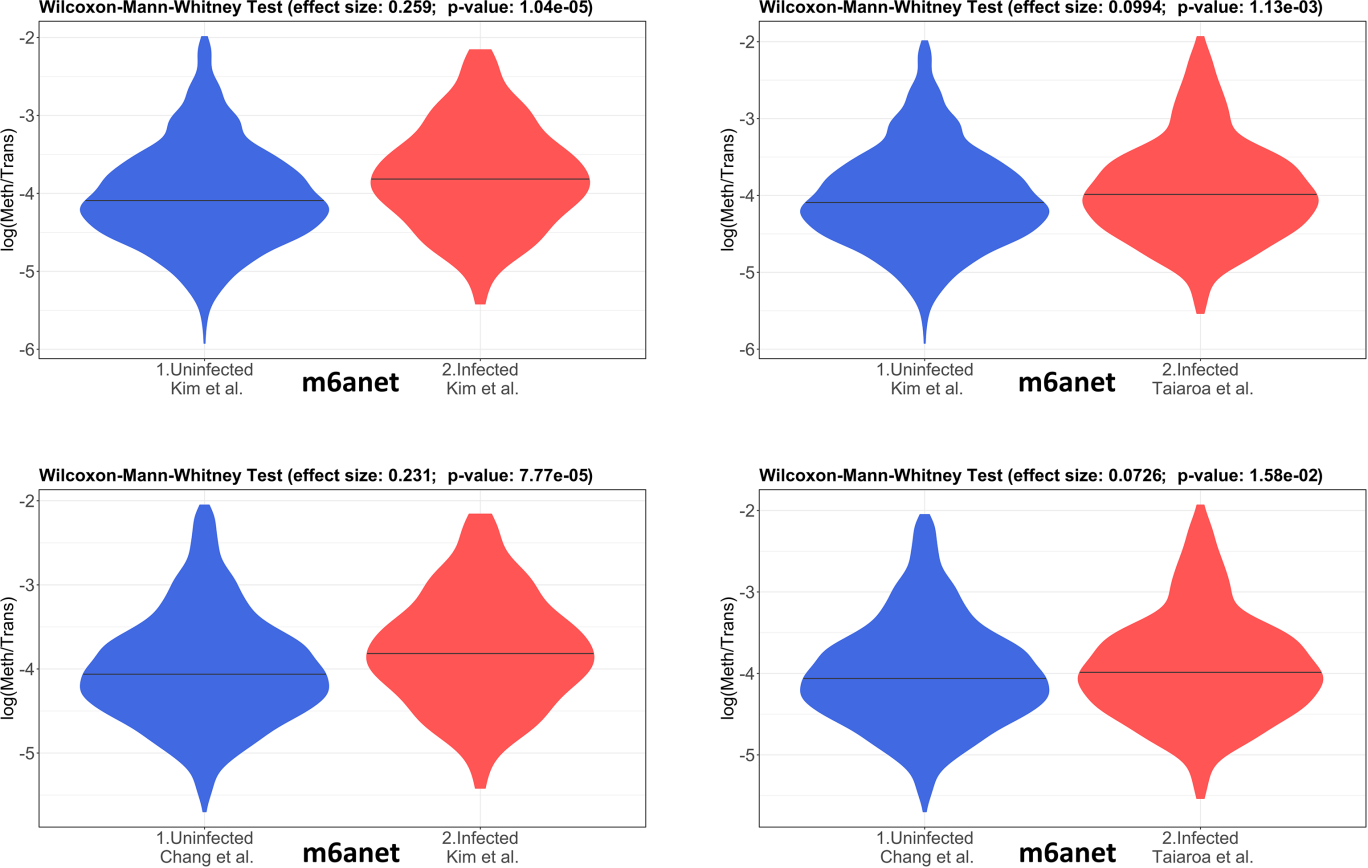
Violin plots of the distributions of differentially methylated transcripts in Uninfected and Infected Vero cell datasets using program m6anet. The areas indicate the data distribution of each sample and the horizontal bars in the middle of the areas indicate the Medians. The Effect size and *p*-values are as obtained by the Wilcoxon-Mann-Whitney test as described in Material and Methods. The abscissas idicate the samples and the ordinates indicate the quantity *S*=log(methylated sites/transcript), which is the logarithm with base 10 of the proportion of methylated sites per transcript, for all transcript types in each dataset.

**Figure 3:**
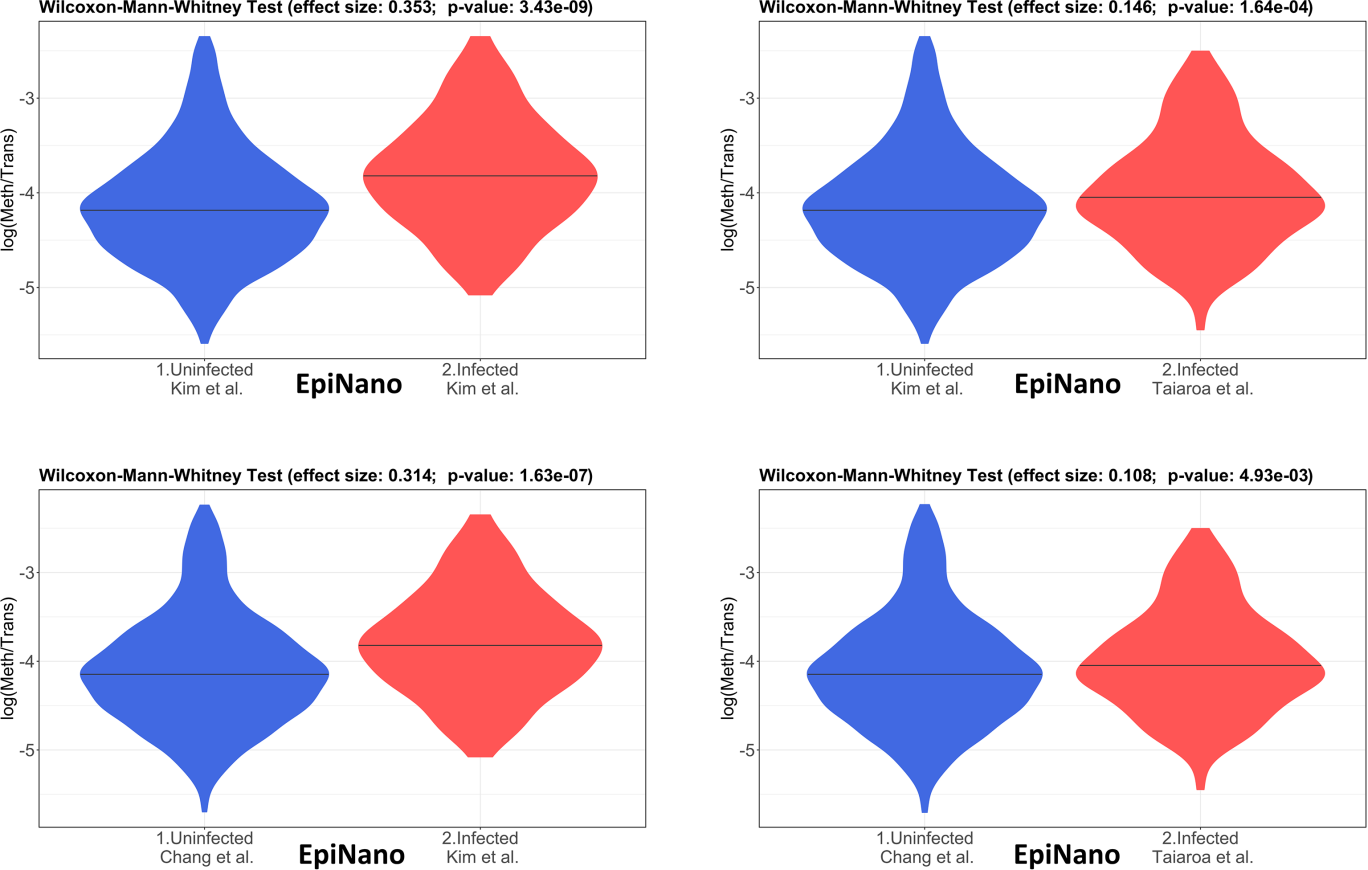
Violin plots of the distributions of differentially methylated transcripts between Uninfected and Infected Vero cell datasets of using program EpiNano. The areas indicate the data distribution of each sample and the horizontal bars in the middle of the areas indicate the Medians. The Effect size and *p*-values are as obtained by the Wilcoxon-Mann-Whitney test as described in Material and Methods. The abscissas idicate the samples and the ordinates indicate the quantity *S*=log(methylated sites/transcript), which is the logarithm with base 10 of the proportion of methylated sites per transcript, for all transcript types in each dataset.

The 4 pairwise comparisons with the WMW test results are shown as violin plots in **Figure 2** for m6A sites detected by program m6anet. All comparisons show higher methylation levels in infected cells with *p*<0.05. Equivalent reults are obtained for the same analysis for m6A sites detected by program EpiNano as shown in **Figure 3**. These results are summarised in **Table 5**. As a control for the method, the WMW test was performed for the comparison of the two uninfected datasets, Kim et al., 2020 (Uninfected Vero) and Chang et al., 2021 (Uninfected Vero 24h), to show that they are statistically equivalent (**Table 5** and **Supplementary Figure S1**). All comparisons between uninfected and infected samples have *p*<0.05 while between uninfected samples *p*>0.05. The lower bounds of the confidence intervals are always >0 in all uninfected versus infected comparisons while in the uninfected versus uninfected control the lower bound is <0. The results obtained with m6anet and EpiNano are equivalent, showing that irrespective of the m6A detection method the increase in m6A methylation is consistently observed (**Table 5** and **Figures 2**, **3**).

**Table 5:** Results of the Wilcoxon-Mann-Whitney test (WMW) for comparison of differentially methylated transcripts between Uninfected (U) and Infected (I) Vero cell datasets using programs m6anet and EpiNano as indicated.

Comparison (WMW)	# Transcripts	Effect Size	Confidence (95%)	*p*-value
**m6anet**				
Kim (U) x Kim (I)	1871 x 137	0.259	0.145 - 0.371	1.04 × 10^-5^
Kim (U) x Taiaroa (I)	1871 x 544	0.099	0.039 - 0.159	1.13 × 10^-3^
Chang (U) x Kim (I)	2372 x 137	0.231	0.117 - 0.344	7.77 × 10^-5^
Chang (U) x Taiaroa (I)	2372 x 544	0.072	0.013 - 0.131	1.58 × 10^-2^
* Kim (U) x Chang (U)	1871 x 2372	0.026	-0.011 - 0.065	0.168
**EpiNano**				
Kim (U) x Kim (I)	1064 x 119	0.353	0.240 - 0.465	3.433 × 10^-9^
Kim (U) x Taiaroa (I)	1064 x 302	0.146	0.069 - 0.222	1.636 × 10^-4^
Chang (U) x Kim (I)	1566 x 119	0.314	0.200 - 0.428	1.632 × 10^-7^
Chang (U) x Taiaroa (I)	1566 x 302	0.107	0.032 - 0.182	4.927 × 10^-3^
* Kim (U) x Chang (U)	1064 x 1566	0.037	-0.008 - 0.084	0.111

(*) Comparison between two Uninfected Vero cell datasets. Datasets are as indicated in Table 1, being Kim (Kim et al., 2020), Taiaroa (Taiaroa et al., 2020) and Chang (Chang et al., 2021).

The analysis of the epitranscriptome of Vero cells as inferred using the EpiNano program using data from (Kim et al., 2020; Taiaroa et al., 2020; Chang et al., 2021) yield resuts equivalent to obtained with m6anet (**Supplementary Tables S18–S22**). However, m6anet detects m6A in DRACH motifs whereas EpiNano detects RRACH, therefore yielding a slightly smaller number of inferred m6A sites due to the reduced degeneracy in the first position of the motif.

### The SARS-CoV-2 m6A epigenome

The epigenome of SARS-CoV-2 from infected Vero cell culture supernatants of (Taiaroa et al., 2020), shows a total of 30 methylated sites, 9 of which are found in the S gene, 4 in the ORF3a, 1 in the M gene, 2 in ORF6, 2 in ORF7a, 2 in ORF7b, and 7 in the N gene (**Supplementary Table S23**). The SARS-CoV-2 epigenome of (Campos et al., 2021) revealed the presence of 20 m6A sites, of which 3 are in the S gene, 4 in the ORF3a, one in the M gene, one in the ORF7a, 2 in the ORF7b, and 6 in the N gene (**Supplementary Table S24**). The samples obtained from the supernatants do not share m6A sites. The SARS-CoV-2 epigenome from infected Calu-3 cells reveals 11 m6A sites in ORF1ab (**Supplementary Table S25**).

### DRACH motif nucleotide biases

**Figure 4:**
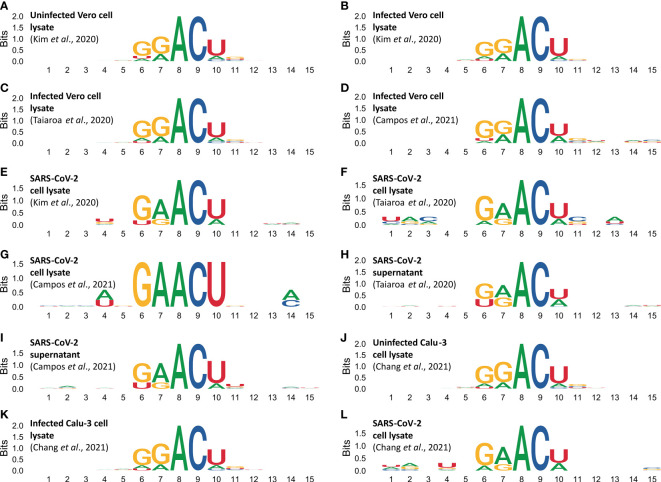
Methylated DRACH motifs reveal different sequence profiles in the cellular epitranscriptome and the viral epigenome. DRACH sequences containing predicted m6a sites (plus 5 nucleotides for each end) were aligned and stacked together to provide an overview of the informational content of methylated regions. Motif profiles in epitranscriptomes were obtained from uninfected Vero cells **(A)**, and from different samples of infected Vero cells **(B–D)**. DRACH profiles in SARS-CoV-2 epigenomes were obtained from different samples of Vero cell lysates **(E–G)**, and from different samples of supernatants **(H, I)**. Motif profiles in epitranscriptomes obtained from uninfected Calu-3 cells **(J)**, infected Calu-3 cells **(K)**. DRACH profiles in SARS-COV-2 epigenome were obtained from infected Calu-3 cells **(L)**. Direct RNA sequencing data were obtained from Kim et al., 2020 **(**in **A, B, E)**, Taiaroa et al., 2020 (in **C, F, H**). Sequencing data obtained by Campos et al., 2021 study are presented in **(D, G, I)**. Data from Chang et al., 2021 on Calu-3 cells **(J–L)**. Numbers in the abscissa indicate the DRACH motif positions (from 6 to 10), the flanking 5’ (from 1 to 5) and 3’ (from 11 to 15). The ordinates indicate the score in Bits as it deviates from the null hypothesis, in other words the stronger the bias, the higher the score. A and C have 100% frequency in positions 8 and 9 of all DRACH motifs analyzed whereas positions with zero or near zero indicate that the four canonical bases are at equilibrium frequency *f_**(N)**_=*0.25 in the same sampling space.

Analysis on sequences flanking m6A sites reveal biases in the DRACH motifs (**Figure 4**). **Figures 4A–D** shows DRACH motifs obtained from Vero cell epitranscriptomes. DRACH motifs of SARS-CoV-2 epigenomes obtained from the lysate of infected Vero cells are shown in **Figures 4E–G** and supernatants in **Figures 4H, I**. DRACH motifs in host cell epitranscriptomes display the signature GGACU, while DRACH in viral epigenomes display the signature GAACU (**Figure 4**). Data from human Calu-3 reveals the same pattern as observed in Vero cells (**Figures 4J–L**).

### Functional enrichment analysis of epitranscriptomes

**Figure 5:**
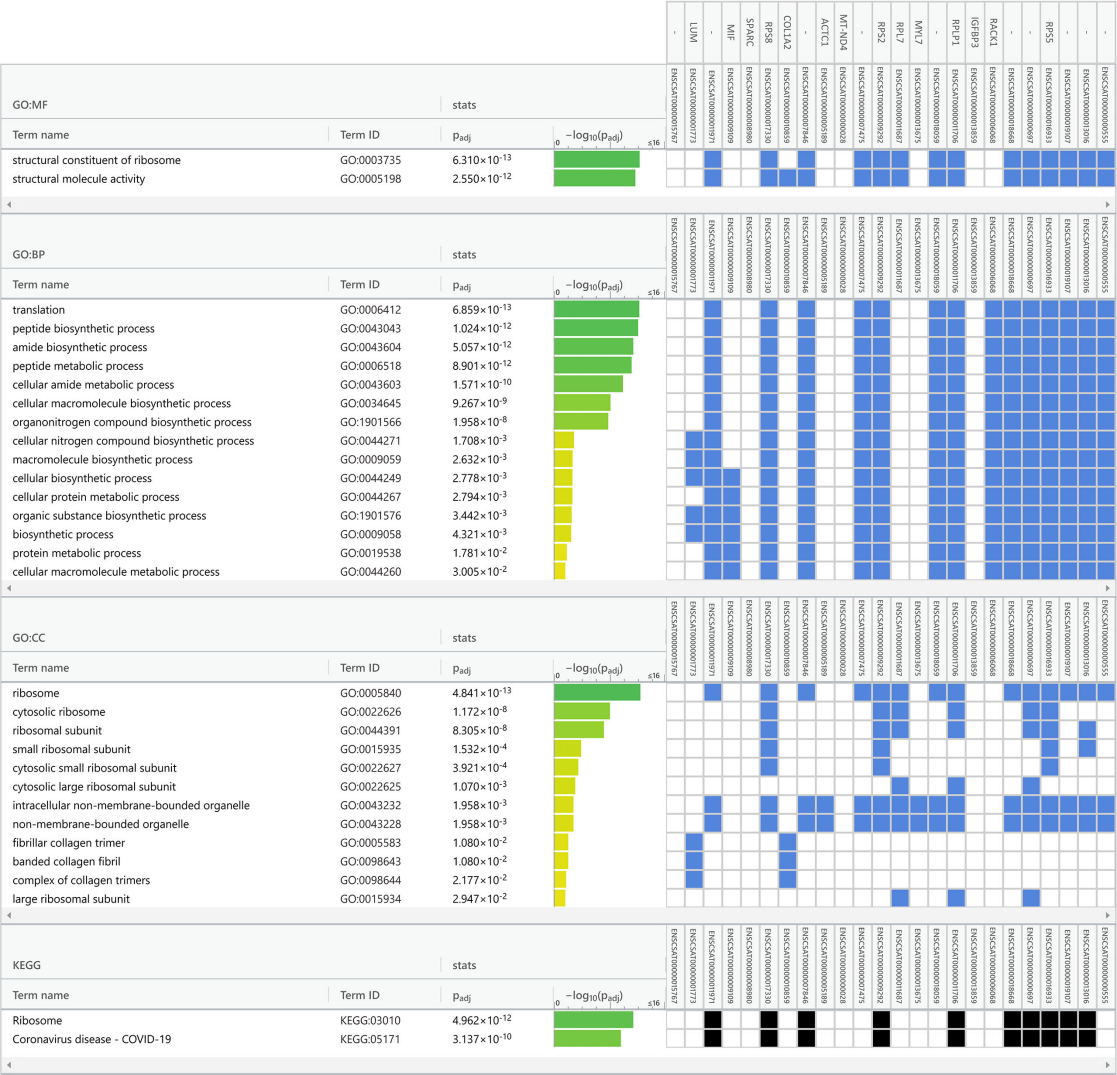
Functional enrichment analysis of the infected Vero cell m6A epitranscriptome (dataset from Campos et al., 2021). A total of 24 transcripts common to infected cells was used in enrichment analysis (**Table 1**), with Gene Ontology and KEGG biological pathways as data sources for overrepresentation. The analysis was performed with default gProfiler web server options, with g:SCS algorithm for computing multiple testing correction for *p*-values. Terms are grouped by data sources (Gene Ontology classifications or KEGG biological pathways). The GO categories are in the left columns, green bars indicate the -log_10_ of the *p*-value, blue and black squares indicate significant positive hits of the transcript IDs (vertical top columns) with GO categories. GO : MF, Molecular Function; GO : BP, Biological Process; GO : CC, Cellular Component and KEGG, Kyoto Encyclopedia of Genes and Genomes.

Data obtained from Vero cell epitranscriptomes were used as inputs for functional enrichment analyses (**Figure 5** and **Supplementary Figures S2, S3**). Among the main biological processes (BP) related to the transcripts listed in **Supplementary Table S2** (sample from Kim et al., 2020), are ‘translation” (GO:0006412), “peptide metabolic process” (GO:0006518), and “peptide biosynthetic process” (GO:0043043) (**Supplementary Figure S2**). Among the KEGG pathways also related to the transcripts listed in **Table 2**, are “Phagosome” (KEGG:04145), “Coronavirus disease – COVID-19” (KEGG:05171), and “Ribosome” (KEGG:0043043) (**Supplementary Figure S2**). Functional enrichment analysis of Vero cell transcriptome from Taiaroa et al., 2020 points to the main BPs, such as “cellular macromolecule metabolic process” (GO:0044260), “peptide metabolic process” (GO:0006518), and “protein metabolic process” (GO:0019538) (**Supplementary Figure S3**). KEGG also pointed as main pathways involved: “Ribosome” (KEGG:03010), “Protein processing in endoplasmic reticulum” (KEGG:04141), and “Cell cycle” (KEGG:04110) (**Supplementary Figure S3**).

Finally, we performed a functional enrichment analysis of Vero cell transcriptome in our sequence data (**Tables 1**, **2**). The main related BPs were “translation” (GO:0006412), “peptide biosynthetic process” (GO:0043043), and “amide biosynthetic process” (GO:0043604) (**Figure 5**. KEGG main pathways involved were: “Ribosome” (KEGG:03010), and “Coronavirus disease – COVID-19” (KEGG:05171) (**Figure 5**).

## Discussion

In the present study we tested the hypothesis that the infection of Vero cells by SARS-CoV-2 affects the m6A methylation patterns of cellular transcripts. For this, the transcriptome of the infected cell was sequenced using the Nanopore direct RNA sequencing method (Oxford Nanopore Technologies, Oxford Science Park, Oxford, UK). Datasets from four studies were compared. One from our group (Campos et al., 2021) and three others from (Kim et al., 2020; Taiaroa et al., 2020; Chang et al., 2021).

Statistical analysis of data here presented revealed that the m6A methylation of cellular RNAs is significantly higher in infected cells as compared to uninfected cells (**Table 5**, **Figures 2**, **3**). This finding is supported by two different m6A detection programs (m6anet and EpiNano) (Liu et al., 2019; Liu et al., 2021a; Hendra et al., 2021). The *p*-values of unifnfected versus infected sample comprarions are always <0.05 and the lower bound of confidence intervals are always >0 which indicates the rubustness of the inference (**Table 5**). The functional enrichment analysis of these datasets revealed an increased methylation of genes involved in translation, peptide and amine metabolism which is consistent with a scenario in which the viral infection reduces the general translational activity of the cell, activation of stress-induced signaling pathways, and employing viral proteins that affect cellular translation and RNA stability to direct the translational machinery towards the synthesis of its own proteins (Nakagawa et al., 2016). The m6A methylation of transcripts involved in the general cellular translation function is consistent with observations that m6A methylation in coding domains slows down translation elongation because m6A leads to ribosome pausing in a codon-specific manner (Choi et al., 2016). However, recent studies have shown multiple roles of m6A in regulating translation and both positive and negative effects of this epitranscriptomic signal on protein synthesis have been reported (Mao et al., 2019). Methylation at different mRNA regions may have distinct functions, therefore it is important to elucidate the local effects of m6A on translation. Here we provided initial data on the general patterns of m6A in cellular transcripts and further studies are necessary to determine the local effects of m6A in individual transcripts.

The quantitave analysis, *via* WMW test, shows that the global m6A methylation is higher in infected Vero cells as compared to uninfected cells (**Figure 2**). Also, results obtained using two different m6A detection programs, m6anet and EpiNano, yield equivalent results (**Figures 2**, **3**). As discussed above it still remains to future work to determine if this global higher m6A methylation is inhibiting or enhancing the translatability of cellular mRNAs. The qualitative analysis, as discussed below, suggests that transcripts of genes involved in translation, peptide and amine metabolism are differentially m6A methylated upon SARS-CoV-2 infection.

The analysis here presented allowed the identification of differentially methylated transcripts and m6A unique sites in the infected cell transcripts (**Tables 3**, **4**) and confirms the general m6A pattern observed with miCLIP and RIP-seq techniques (Liu et al., 2021b). However, it must be noted that this study (Liu et al., 2021b) used RIP-seq which do not have a 1 nucleotide resolution and miCLIP, athough claiming a 1 nucleotide resolution depends on antibody crosslink and cDNA sequencing. Among the three datasets here analyzed we decided to use the sample set of (Kim et al., 2020) for these analyses because this dataset contains the largest number of mapped reads (**Table 1**). This dataset allowed the identification of differentially methylated transcripts in the SARS-CoV-2 infected Vero cells (**Table 3**). This analysis revealed that at least 55 sites, distributed in 21 known genes, are differentially methylated. The majority of transcripts show a reduced m6A methylation upon infection such as *TMED2*, while a few show increased methylation, such as the proto-oncogene *JUNB*, a key transcriptional modulator of macrophage activation (Fontana et al., 2015) and the immediate early response *IER3*, involved in cellular stress response and inflammation (Arlt and Schäfer, 2011). Other interesting transcripts revealed in this analysis are Tensin 3 (*TNS3*), *NUAK2*, and *METTL9* (detailed below). Also, this same dataset allowed the identification of m6A sites unique to infected Vero cell transcripts (**Table 4**). This analysis revealed 47 sites distributed in 37 known genes with attention to the kinase *NUAK2*, a critical target in liver cancer (Yuan et al., 2018), Tensin 3 (*TNS3*), a SH2 domain protein that contributes to tumorigenesis and metastasis (Qian et al., 2009), Ras member B homolog (*RHOB*), a member of the Rho GTP-binding protein family (Wennerberg and Der, 2004) and *METTL9*, a methyltransferase that mediates pervasive 1-methylhistidine modification in mammalian proteomes (Davydova et al., 2021).

The transcriptome-wide analysis shows very strong nucleotide biases in DRACH motifs of cellular transcripts, which use the signature GGACU, both in Vero cells and Calu-3 cells (**Figures 4A–D, J, K**), whereas in viral RNAs the signature is GAACU (**Figures 4E–I, L**). In Influenza virus it has been shown that the DRACH motif biases are much less significant, and the Influenza virus signature is AAACN with frequencies A=0.50, G-0.25 and U=0.25 in the first position, A=G=0.50 in the second position and A=33.3, C=33.3, U=0.25 and G=0.83 in the fifth position (Bayoumi and Munir, 2021). In positions 3 and 4 respectively, A and C are 100%. This is substantially different from what we found in SARS-CoV-2 (**Figures 4E–I, L**). Moreover, the Influenza virus study was based on cDNA sequences while our observations are based on direct RNA sequencing. Our data show that the sequence preference for methylation in the viral genome is different from the cellular transcripts. This is consistent with the fact that SARS-CoV-2 is a recent primate pathogen. We hypothesize that this virus might be undergoing an adaptive process that would result in the adjustment of its m6A methylation pattern to match those of the cellular transcripts because both use host encoded writer, reader and eraser enzymes (Li et al., 2021).

It is important to note that the direct RNA sequencing has been validated by orthogonal methods to identify modified bases as revealed by the comparison with liquid chromatography-tandem mass spectrometry and methylated RNA immunoprecipitation sequencing (MeRIP-seq) (Li et al., 2021). Therefore, results obtained with direct RNA sequencing, and the downstream bioinformatic pipelines, readily identify modified bases, particularly methylated modifications confirmed by the above-mentioned techniques.

Functional enrichment analysis is a set of statistical methods to extract biological information from omics data in terms of functional categories. These methods are widely used for the analysis of gene and protein lists and regulatory elements (Garcia-Moreno et al., 2022). Taken together our results on functional enrichment analysis (**Figure 5** and **Supplementary Figures S2**, **S3**), differential methylation (**Table 3**) and unique m6A in infected cells (**Table 4**) indicate that the cell response to viral infection not only changes the levels of mRNAs, as previously shown (Wyler et al., 2021), but also its epitranscriptional pattern.

Here, the epitranscriptomics of the Vero cell was studied because of its widespread use for Coronavirus isolation and propagation *in vitro*. This cell line is derived from the African Green Monkey, or vervet (*Chlorocebus sabaeus*) and therefore is a model, or an approximation, for the human infection pattern (Jasinska et al., 2013; Warren et al., 2015). Although the genomes of great apes, including humans, differ from monkeys by 7% it must be noted that in genomes on the order of 7 billion bases (diploid genomes) 93% identity means a difference of 490 million substitutions (Rhesus Macaque Genome Sequencing and Analysis Consortium et al., 2007). Therefore, results obtained from non-human primates must be taken in perspective and cannot be extrapolated *in limine* for humans, based only on a superficial notion of similarity (Jasinska et al., 2013; Woolsey et al., 2021). Consequently, future experiments on the epitranscriptome of human cell lines infected with SARS-CoV-2 are essential for a proper understanding of the human cellular response in the context of SARS-CoV-2 infection.

## Data availability statement

The data presented in this study are deposited in the National Center for Biotechnology Information (NCBI) as BioProject ID: PRJNA861323, BioSamples: SAMN29911639 and SAMN29911623.

## Author contributions

MB, JC and LJ conceptualized the work. MB, JM and CB contributed with wet lab data and results. MB, JC, GVA and FA performed the computational analysis and validation. MB, JC and FA wrote the manuscript. All authors have read, edited and agreed to the published version of the manuscript.

## Funding

Fundação de Amparo à Pesquisa do Estado de São Paulo (FAPESP, Brazil) grant 2020/08943-5 to LJ, MB, FA; Conselho Nacional de Desenvolvimento Científico e Tecnológico (CNPq, Brazil) grant 405691/2018-1 to CB; Conselho Nacional de Desenvolvimento Científico e Tecnológico (CNPq, Brazil) grant 303912/2017-0 to MB; JC received a postdoctoral fellowship from Fundação de Amparo à Pesquisa do Estado de São Paulo (FAPESP, Brazil) grant 2021/13111-1.

## Acknowledgments

The authors are deeply thankful to Prof. Fulvio A. Scorza, the director of *Escola Paulista de Medicina* at UNIFESP, for his inspirational leadership and committed support to the Center for Medical Bioinformatics at the Federal University of São Paulo (UNIFESP), Brazil.

## Conflict of interest

The authors declare that the research was conducted in the absence of any commercial or financial relationships that could be construed as a potential conflict of interest.

## Publisher’s note

All claims expressed in this article are solely those of the authors and do not necessarily represent those of their affiliated organizations, or those of the publisher, the editors and the reviewers. Any product that may be evaluated in this article, or claim that may be made by its manufacturer, is not guaranteed or endorsed by the publisher.
