# Supplementary material for: The epitranscriptome of Vero cells infected with SARS-CoV-2 assessed by direct RNA sequencing reveals m6A pattern changes and DRACH motif biases in viral and cellular RNAs
Source: Front Cell Infect Microbiol. 2022 Aug 16;12:906578. doi: 10.3389/fcimb.2022.906578 (PMC9425070; doi:10.3389/fcimb.2022.906578)
Supplement: Supplementary file 1 [file DataSheet_1.zip › Supplementary Materials/Supplementary Figures Legends.docx]

**Supplementary Figures Legends**

**The Supplementary Figures are in separate High Resolution scalable png files to facilitate visualization of details.**

**Supplementary Figure S1 |** Violin Plots of differentially methylated transcripts of 2 Uninfected Vero cell datasets compared by A) m6anet and B) EpiNano. The horizontal bars indicate the medians.

**Supplementary Figure S2 |** Functional enrichment analysis of the epitranscriptome of the infected Vero cell (sample from Kim et al., 2020 study). A total of 137 transcripts common to infected cells was used in enrichment analysis (Table 2), with Gene Ontology and KEGG biological pathways as data sources for overrepresentation. The analysis was performed with default gProfiler web server options, with g:SCS algorithm for computing multiple testing correction for p-values. Terms are grouped by data sources (Gene Ontology classifications or KEGG biological pathways).

**Supplementary Figure S3 |** Functional enrichment analysis of the epitranscriptome of the infected Vero cell (sample from Taiaroa et al., 2020 study). A total of 544 transcripts common to infected cells was used in enrichment analysis (Table 3), with Gene Ontology and KEGG biological pathways as data sources for overrepresentation. The analysis was performed with default gProfiler web server options, with g:SCS algorithm for computing multiple testing correction for p-values. Terms are grouped by data sources (Gene Ontology classifications or KEGG biological pathways).
