# Supplementary figures and images for: The epitranscriptome of Vero cells infected with SARS-CoV-2 assessed by direct RNA sequencing reveals m6A pattern changes and DRACH motif biases in viral and cellular RNAs
Source: Front Cell Infect Microbiol. 2022 Aug 16;12:906578. doi: 10.3389/fcimb.2022.906578 (PMC9425070; doi:10.3389/fcimb.2022.906578)

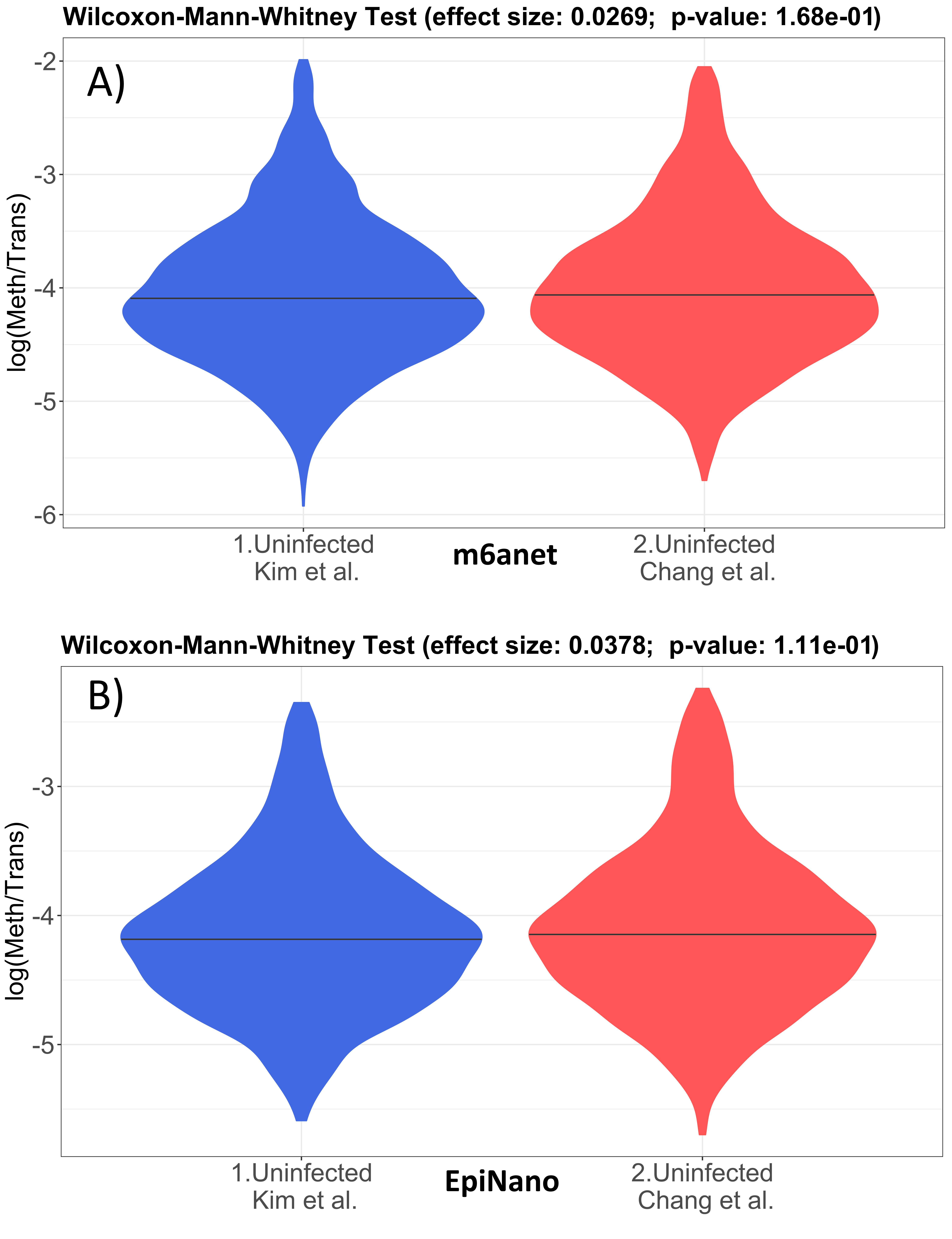

Supplement: Supplementary file 1 [file DataSheet_1.zip › Supplementary Materials/Supplementary Figure S1. Differentially methylated transcripts - 2 Uninfected Vero cell datasets - m6anet.png]
